# Exercise Physiology Across the Lifespan in Cystic Fibrosis

**DOI:** 10.3389/fphys.2019.01382

**Published:** 2019-11-05

**Authors:** Ren-Jay Shei, Kelly A. Mackintosh, Jacelyn E. Peabody Lever, Melitta A. McNarry, Stefanie Krick

**Affiliations:** ^1^Division of Pulmonary, Allergy, and Critical Care Medicine, Department of Medicine, The University of Alabama at Birmingham, Birmingham, AL, United States; ^2^Gregory Fleming James Cystic Fibrosis Research Center, The University of Alabama at Birmingham, Birmingham, AL, United States; ^3^Applied Sports, Technology, Exercise and Medicine Research Centre, College of Engineering, Swansea University, Swansea, United Kingdom; ^4^Medical Scientist Training Program, The University of Alabama at Birmingham, Birmingham, AL, United States

**Keywords:** cystic fibrosis, pediatric, exercise capacity, aging, exercise prescription

## Abstract

Cystic fibrosis (CF), a severe life-limiting disease, is associated with multi-organ pathologies that contribute to a reduced exercise capacity. At present, the impact of, and interaction between, disease progression and other age-related physiological changes in CF on exercise capacity from child- to adult-hood is poorly understood. Indeed, the influences of disease progression and aging are inherently linked, leading to increasingly complex interactions. Thus, when interpreting age-related differences in exercise tolerance and devising exercise-based therapies for those with CF, it is critical to consider age-specific factors. Specifically, changes in lung function, chronic airway colonization by increasingly pathogenic and drug-resistant bacteria, the frequency and severity of pulmonary exacerbations, endocrine comorbidities, nutrition-related factors, and CFTR (cystic fibrosis transmembrane conductance regulator protein) modulator therapy, duration, and age of onset are important to consider. Accounting for how these factors ultimately influence the ability to exercise is central to understanding exercise impairments in individuals with CF, especially as the expected lifespan with CF continues to increase with advancements in therapies. Further studies are required that account for these factors and the changing landscape of CF in order to better understand *how* the evolution of CF disease impacts exercise (in)tolerance across the lifespan and thereby identify appropriate intervention targets and strategies.

## Introduction and Overview of Cystic Fibrosis

Cystic fibrosis (CF) is the most common genetic disease in the Caucasian population, caused by mutations in the cystic fibrosis transmembrane conductance regulator (*CFTR*) gene (Rowe et al., 2005, 2016; Cutting, 2015; Ratjen et al., 2015; Elborn, 2016; Farrell et al., 2017). CF is a multisystem disease affecting the pulmonary, gastrointestinal (GI), and reproductive systems, thereby resulting in increased morbidity and mortality (Rowe et al., 2005, 2016; Ratjen et al., 2015; Stoltz et al., 2015; Elborn, 2016). Defects in CFTR result in airway dehydration and the production of hyper-viscous and acidic mucus, which contributes to defective mucociliary clearance (Fahy and Dickey, 2010; Peabody et al., 2018; Shei et al., 2018). As a consequence, the airways are prone to chronic inflammation and recurrent infection, leading to a vicious cycle that causes progressive, irreversible lung damage and airway obstruction. The resulting pulmonary disease, in combination with a host of other factors including malnutrition (due to exocrine and endocrine pancreatic insufficiency), physical inactivity, and intrinsic muscle abnormalities, contribute to exercise intolerance in people with CF (Marcotte et al., 1986b; Cox and Holland, 2017; Gruet et al., 2017; Urquhart and Saynor, 2018).

**FIGURE 1:**
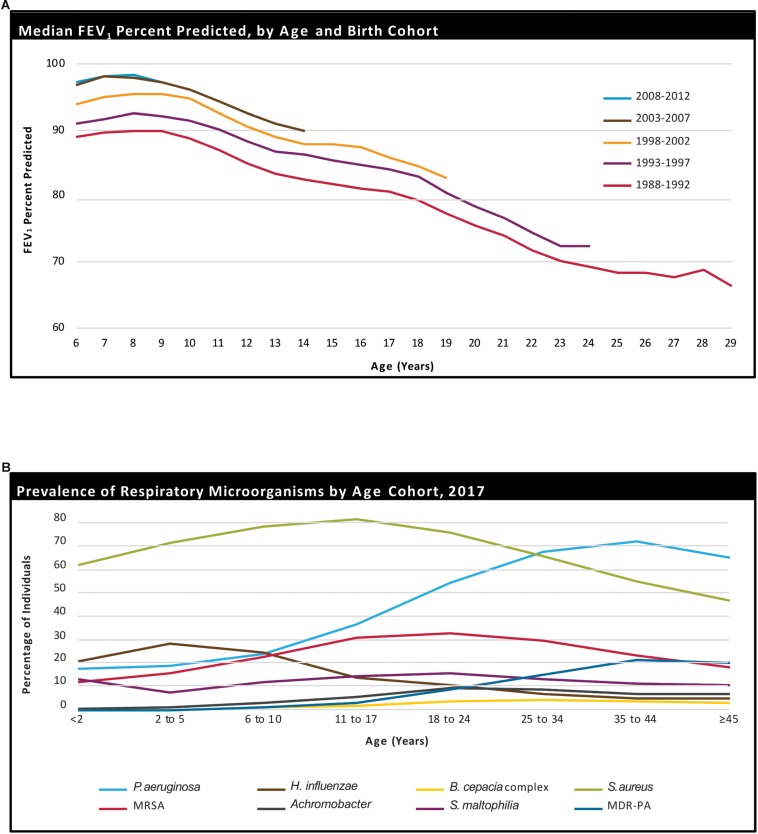
Age-related decline in percent-predicted forced expiratory volume in 1 s (ppFEV_1_) in people with CF, stratified by birth cohort **(A)**. Prevalence of respiratory microorganisms by age cohort **(B)**. Reprinted with permission from Cystic Fibrosis Foundation (2017).

Regular airway clearance and inhaled antibiotic therapy, in combination with the recent development of highly effective CFTR modulator therapies, have greatly extended the life expectancy of people living with CF, allowing these patients to live into, or indeed beyond, their fifth or sixth decade (Solomon et al., 2015; De Boeck and Amaral, 2016; Quon and Rowe, 2016; De Boeck and Davies, 2017; Burgener and Moss, 2018; McElvaney et al., 2018; Rubin, 2018; West and Flume, 2018). This increased expected lifespan has highlighted the need to better understand the evolution of people with CF as they reach ages that were previously impossible or, at best, improbable. Indeed, with age, secondary co-morbidities become more prominent and prevalent. Specifically, co-morbidities such as chronic infections from an ever-changing spectrum of pathogens, some of which may become multi-drug resistant [i.e., *Pseudomonas aeruginosa* (*PsA*), *Burkholderia cepacia* (*B. cepacia*), and atypical mycobacteria, Figure 1A], and more frequent and severe pulmonary exacerbations lead to a progressive lung function decline and may, ultimately, increase mortality (Rowe et al., 2005, 2016; Ratjen et al., 2015; Elborn, 2016). Furthermore, this “aging CF population” has shown an increased incidence of CF-related diabetes (CFRD), low bone mineral density, and endothelial dysfunction due to chronic inflammation. In addition to their system-specific effects, each of these co-morbidities have deleterious consequences for quality of life (QoL) and aerobic fitness. Given the high prognostic value of aerobic fitness for mortality and QoL (Nixon et al., 1992; Moorcroft et al., 1997; Pianosi et al., 2005; Ward et al., 2013; Vendrusculo et al., 2018), understanding *how* exercise capacity changes, as well as the impact of disease progression, as people with CF age, may aid in developing appropriate, individually tailored exercise recommendations, in improving adherence, and, ultimately, in engendering better health outcomes in this population.

While it has long been established that children are not simply “mini-adults,” this is particularly pertinent in clinical populations in which aging occurs concomitantly, and potentially interactively, with, but nonetheless distinctly from, disease progression. Despite the importance of this distinction, the majority of research has failed to account for the influence of age, as well as the process and rate of aging, leading to potentially misleading conclusions regarding the disease progression itself. Indeed, comparisons between children and adults with CF are further compounded by the very different treatment strategies used in each age group – the treatment currently received by children is likely to significantly alter the course of their disease progression, and their experience of it, compared to that of those who are now adults. Therefore, changes in disease pathology, co-morbidities, disease complications, and treatment strategies as patients with CF age must be carefully assessed when designing, conducting and interpreting exercise studies in CF.

## Exercise Capacity in CF

**FIGURE 2:**
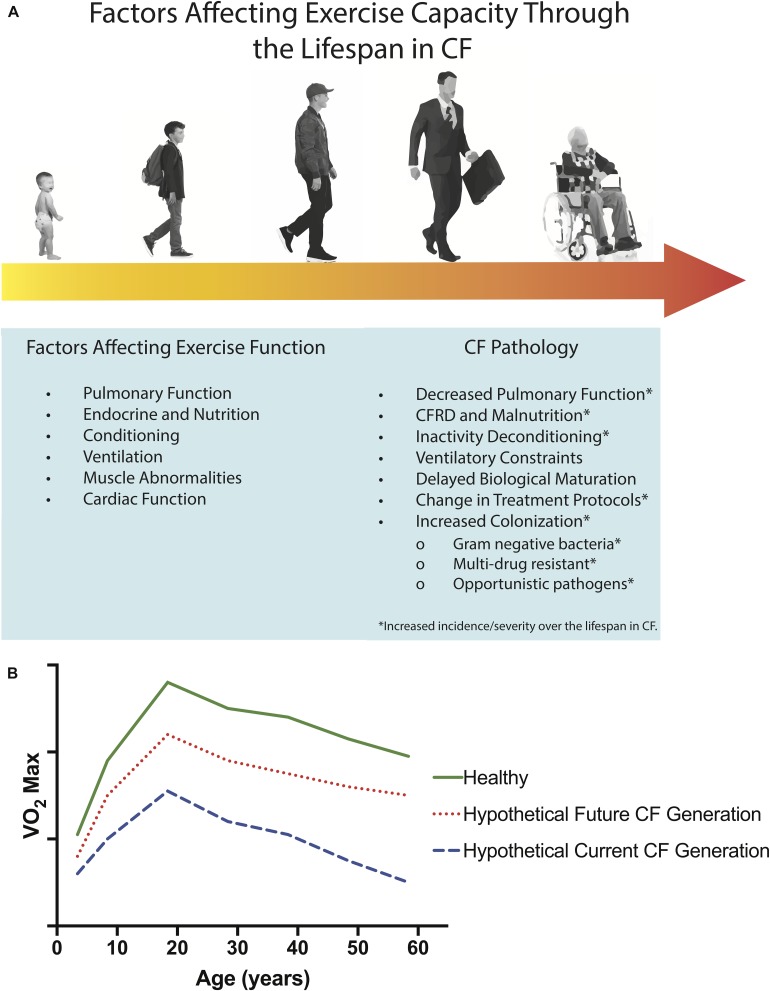
Determinants of exercise dysfunction in CF and age-related factors which may affect exercise capacity through the lifespan in CF **(A)**. Airways in CF may be altered in structure from fetal development or from destructive events, leading to airway malacia. The incidence and severity of factors involved in CF Pathology increase over the lifespan of people with CF, as indicated by the progression from light yellow to dark orange across the lifespan in **(A)**. Projected age-related decline in aerobic capacity (i.e., $\dot{V}$O_2__*max*_) for healthy populations (solid green line, adapted from Shvartz and Reibold (1990) and Booth and Zwetsloot (2010), and hypothetical age-related declines in aerobic fitness for current (dashed blue line) and future (dotted red line) CF populations **(B)**. It is possible that with early initiation of CFTR modulator therapy and effective disease management, that in the future, individuals with CF may possess aerobic capacities more similar to healthy controls than individuals with CF today.

Deficits in exercise capacity (i.e., the maximum amount of physical exertion that a patient can sustain; (Goldstein, 1990) in those with CF result from a combination of factors, including, but not limited to, ventilatory dysfunction, changes in nutritional status, abnormalities in peripheral muscles (i.e., muscle weakness and putative metabolic abnormalities), cardiac constraint, and disease-related deconditioning (Figure 2A). The mechanisms of exercise limitation in CF have been reviewed elsewhere (Hulzebos et al., 2015). Briefly, ventilatory dysfunction in CF may contribute to exercise intolerance through deleterious changes in lung function, dead space ventilation, respiratory muscle function, ventilatory reserve, and ventilatory control. Together, these ventilatory constraints may limit exercise tolerance, particularly in more severe disease states. Specific to ventilatory dysfunction, exercise-induced hypoxemia may be more prevalent in CF, at least in part due to ventilation-perfusion mismatching secondary to increases in physiologic dead space and intrapulmonary arterio-venous shunting (Coffey et al., 1991). Nutritional status also plays an important role in determining exercise limitation in CF, particularly in those who are malnourished. Indeed, malnutrition predisposes individuals with CF to loss of both muscle mass and body fat, impaired diaphragmatic performance, and negatively affects cardiac function (Marcotte et al., 1986a; Lands et al., 1992a, b). Finally, muscle abnormalities including muscle weakness, mitochondrial dysfunction, and altered muscle metabolism may also contribute to exercise intolerance in CF (de Meer et al., 1995; Meer et al., 1999; Divangahi et al., 2009; Lamhonwah et al., 2010; Wells et al., 2011; Gruet et al., 2016; Werkman et al., 2016; Gruet et al., 2017). Whilst the mechanisms underpinning exercise limitation in those with CF are complex and interdependent (Schöni and Casaulta-Aebischer, 2000; Divangahi et al., 2009; Lamhonwah et al., 2010; Pastré et al., 2014; Hulzebos et al., 2015; Jiang et al., 2016; Gruet et al., 2017), it has been postulated that age-related progressions in CF disease severity may be integral to the annual decrements typically observed.

## Effects of Aging on Exercise in CF

Despite evidence that exercise in people with CF improves aerobic capacity and thereby reduces mortality (Radtke et al., 2017), one of the most significant challenges is ensuring engagement in habitual physical activity (White et al., 2007; Myers, 2009). Indeed, engagement from a young age is imperative as not only is this likely to attenuate the decline in fitness and function and to promote the level from which this decline occurs, but importantly, behaviors established during childhood track into adulthood (Dishman et al., 1985). This therefore highlights the need to instill healthy behaviors at an early age. Recent case series reports regarding the influence of Orkambi^®^, one of the CFTR modulator combination therapies, on daily physical activity and exercise tolerance over a 2 year period are highly encouraging (Savi et al., 2019), especially when considered in conjunction with reports of improvements in peak oxygen consumption ($\dot{V}$O_2__*peak*_) and percent predicted forced expiratory volume in 1 s (ppFEV_1_; Hatziagorou et al., 2018; Philipsen and Pressler, 2018). Thus, as these therapies become more common and are able to be initiated at younger ages, exercise capacity may be preserved and/or improved in individuals with CF across their lifespan. The question remains, nonetheless, whether these individuals will be able to exercise normally or will still need individually targeted exercise prescriptions due to a sub-normal exercise capacity.

Children and adults with CF have been shown to have a significantly lower $\dot{V}$O_2__*peak*_, lower gas-exchange threshold, reduced work capacity, and reduced oxygen uptake efficiency compared to their healthy peers (Pouliou et al., 2001; Perpati et al., 2010; Tomlinson et al., 2018). Similarly, a reduced time to exhaustion during ramp testing (Saynor et al., 2016), impaired blood flow regulation, and an exaggerated oxidative stress and slower oxygen uptake response to submaximal exercise have been shown in children with CF (Tucker et al., 2018). While these impairments in exercise capacity are evident from a young age in CF and persist into adulthood, it is not yet known whether the differences between CF and healthy controls may become more pronounced with age. Indeed, no studies have sought to directly compare the exercise capacity of children and adults with CF. However, it is pertinent to note that such comparisons may be complicated by changes in pharmacological and non-pharmacological treatment strategies over the last few decades. Therefore, longitudinal studies that account for patient variations in treatment strategies are required; the increasing availability and utilization of cardiopulmonary exercise testing during annual reviews will be instrumental in capturing this information. In healthy populations, maximal oxygen consumption $\dot{V}$O_2__*max*_ is known to decline with age, although regular exercise can slow this decline (Figure 2B – including hypothetical projected age-related declines for CF populations; Hagberg, 1987; Hawkins and Wiswell, 2003). The magnitude and rate of decline in $\dot{V}$O_2__*max*_ in those with CF due to an accelerated ageing process still remains to be disentangled from the influence of CF *per se*.

### Bacterial Colonization

One of the most prominent differences between children and adults with CF is the degree and consistency of pulmonary colonization. Patient Registry data from the US Cystic Fibrosis Foundation shows that children under the age of 17 years tend to be colonized predominantly with *Staphylococcus aureus* (*S. aureus*), methicillin-resistant *S. aureus*, and *Haemophilus influenzae*, whilst adults have a larger percentage of *PsA* and an increase in the proportion of *Stenotrophomonas maltophilia* and *B. cepacia* (Figure 1B; Parkins and Floto, 2015; Cystic Fibrosis Foundation, 2017). Recurrent and increasingly persistent respiratory infections in youth, along with viral and fungal infections, contribute to repeated pulmonary damage, and as the disease progresses, these people with CF become more susceptible to colonization and infection with gram-negative bacteria, including multidrug resistant *PsA* (Elborn, 2016; Winstanley et al., 2016).

Recurrent and difficult to treat pulmonary infections are major determinants of progressive pulmonary decline over the lifespan, which coincides with progressive loss of exercise capacity (Rowe et al., 2005, 2016; Ratjen et al., 2015; Elborn, 2016; Radtke et al., 2017). Deteriorating lung function itself can contribute to airflow limitation, ventilatory-perfusion mismatch, predisposition to desaturation during exercise, and respiratory muscle weakness, all of which have profound impacts on exercise capacity. However, despite impaired exercise capacity being a common characteristic of CF, the underpinning physiological mechanisms are generally unknown. It is therefore unsurprising that studies and reviews incorporating children, adolescents, and adults with CF have failed to consider how age mediates, or impacts, exercise capacity (Radtke et al., 2017; Abdelbasset et al., 2018). Nonetheless, recent research has shown that even young children, with fewer exacerbations, had a lower exercise capacity in comparison to healthy counterparts. Indeed, Abdelbasset et al. (2018) found that CF children’s $\dot{V}$O_2__*peak*_ was significantly correlated with quadriceps strength and endurance. Therefore, the function of the peripheral muscles may play a significant role in the decreased exercise capacity in children with CF (Ferrari et al., 2015; Gruet et al., 2017). In adults with CF, the strength of the quadriceps has been associated with aerobic capacity and lung function, with those with airway obstruction unable to undertake continuous exercise due to lower extremity fatigue (Moorcroft et al., 2005). Across the age spectrum, Troosters et al. (2009) reported reduced skeletal muscle strength and endurance of those with CF, which was associated with decreased exercise capacity and subsequent clinical complications. This has been postulated to be associated with early neuromuscular activity deteriorations in the quadriceps, as observed following high-intensity aerobic exercise in chronic obstructive pulmonary disease (COPD) (Mador et al., 2000), although it should be noted that the pathophysiologic basis for this may differ between COPD and CF. Therefore, when designing, conducting, and evaluating exercise physiology studies in CF, it is important to not only account for age, but the associated changes in colonization and systemic inflammation, which can be markedly different across age groups in CF.

### Frequency of Pulmonary Exacerbations and Hospitalization

Progression of CF lung disease with age contributes substantially to the more frequent occurrence of pulmonary exacerbations and consequent hospitalizations. Importantly, during hospitalizations for acute pulmonary exacerbations, physical activity is reduced or even absent (Ward et al., 2013). This may be due, at least in part, to a lack of access to exercise facilities (due to infection control measures; (Saiman et al., 2014), in addition to the pulmonary symptoms and associated treatments resulting from the exacerbation itself. When aerobic exercise is conducted during hospital admissions in children with CF, a substantially improved $\dot{V}$O_2__*max*_ has been reported at discharge (Selvadurai et al., 2002), highlighting the importance of maintaining physical activity, irrespective of form (i.e., exercise), during hospitalization. The increased frequency of hospitalizations in adulthood likely contributes to declining physical activity during, and immediately following, these episodes. Any decline in physical activity levels could impact on the ability to complete activities of daily living, accelerate their decline in pulmonary function, and deteriorate QoL in those with CF (Wilkes et al., 2009; Dwyer et al., 2011).

### Nutrition

Nutrition is increasingly recognized as a key determinant of physical, mental and social health across the lifespan, especially for people with CF. Because CFTR is abundantly expressed in the exocrine pancreatic and biliary secretory system, mutations in *CFTR* can result in mucus obstruction in these organs and consequent exocrine pancreatic insufficiency (Rowe et al., 2005, 2016; Ratjen et al., 2015; Elborn, 2016). Thus, prominent GI manifestations of CF disease are evident, resulting in malabsorption of fat, secondary nutritional loss, malabsorption and deficiency of fat-soluble vitamins, chronic gastro-esophageal reflux, and susceptibility to recurrent small bowel obstruction. Progressive damage to the endocrine pancreas, resulting from protein accumulation (consequent to CFTR dysfunction) and precipitation within the pancreatic ducts that causes ductal destruction and ischemic damage (Laguna et al., 2010; Gibson-Corley et al., 2016), leads to the occurrence of CFRD in a majority of individuals with CF, which is clinically distinct from traditional Type 1 and Type 2 diabetes mellitus in non-CF populations. The prevalence of CFRD increases across the lifespan, from 2% in children to 19% in adolescents and 50% in adults (≥18 years) living with CF (Moran et al., 2010; Lewis et al., 2015); over 90% of pancreatic insufficient CF patients have CFRD by approximately 50 years old. Thus, CFRD is an important comorbidity, which becomes more prevalent with age and may contribute to the loss of exercise capacity. Exercise training has been shown to improve glycemic control in CF (Beaudoin et al., 2017), highlighting nutrition not only as a determinant of exercise capacity but also a potential area of benefit from regular exercise training.

CF-related diabetes is highly relevant given that malnutrition, weight loss, and lean muscle mass loss all contribute to exercise intolerance. Moreover, CFRD also negatively impacts pulmonary function and, consequently, morbidity and mortality (Chase et al., 1979; Milla et al., 2000; Pencharz and Durie, 2000; Borowitz et al., 2002; Sinaasappel et al., 2002; Milla, 2007; Moran et al., 2010; Lewis et al., 2015; Wolfe and Collins, 2017; Collins, 2018; Rozga and Handu, 2019). Lung bacterial clearance is negatively affected by hyperglycemia with hyperglycemia contributing to increases in inflammation and infection (Brennan et al., 2007; Hunt et al., 2014), which may partially account for these CFRD sequelae. CFRD and malnutrition may affect exercise capacity most directly by predisposing patients toward lean muscle mass loss, atrophy, and cachexia, particularly when nutritional needs are not adequately met. Furthermore, secondary morbidities associated with uncontrolled CFRD, such as neuropathy, nephropathy and retinopathy, can have additional deleterious effects on exercise capacity and QoL (Rosenecker et al., 2001; Laguna et al., 2010). Specifically, these deleterious effects are mediated by factors including, but not limited to: loss of balance, coordination, reflexes, and muscle weakness resulting from diabetic neuropathy; impaired blood pressure control, nausea, and vomiting, fatigue, peripheral edema and potentially progression to renal failure resulting from diabetic nephropathy; and blurred vision, glaucoma, cataracts, and macular edema resulting from diabetic retinopathy. Furthermore, underweight patients (BMI < 18 kg m^–2^) can experience a sustained catabolic state resulting, in part, from prolonged malnutrition, ultimately resulting in chronic weight loss and difficulty maintaining or gaining weight, which negatively affects lung function. This clinical presentation can adversely affect lean muscle mass, contribute to muscular atrophy and cachexia, and inadequate bioenergetic stores to support exercise. Indeed, malnutrition is increasingly observed throughout maturity, with growing recognition that a low-fat free mass may be hidden by a normal, or elevated, BMI. Therefore, proper management of nutrition in CF (and of CFRD in individuals who present with it) is an important method to preserve or even enhance, exercise capacity. At present, however, exercise nutrition guidelines specific to the CF population are not available, and the most recent clinical nutrition guidelines (Turck et al., 2016; Rozga and Handu, 2019) do not address exercise nutrition in CF. We therefore suggest that future research seek to address the unique nutritional needs of individuals with CF during exercise.

While it is still important to consider the nutritional status in pediatric individuals with CF, the acute and chronic effects of malnutrition become more evident with age partially due to increasing chronic inflammation and infections, and therefore require more interventions to manage. Such interventions may include the use of percutaneous endoscopic gastric feeding tubes, peripherally inserted central catheters and/or ports, all of which may represent barriers to exercise. For example, anecdotal evidence suggests that patients with feeding tubes experience pain in the abdominal muscles during workouts, which may prevent them from engaging in core-strengthening exercises, or dynamic exercises which require significant activation of the core muscles. It is also important to consider the socioecological factors associated with such interventions, especially in young pre-pubertal and pubertal CF populations, which further predispose them to avoid physical activity.

### Biological Maturation

There is contradicting evidence regarding whether biological maturation, most commonly assessed through age at peak height velocity (PHV) and age at menarche, is delayed in youth with CF (Johannesson et al., 1997; Aswani et al., 2003; Bournez et al., 2012; Scaparrotta et al., 2012; Sands et al., 2015). Specifically, while the rate of PHV and final height are suggested to be lower in people with CF compared to their healthy peers, it is still equivocal whether the age of onset of puberty is also different in CF. Alterations in biological maturation could be the result of nutritional factors, the use of corticosteroids, and differences in sex hormone secretion (e.g., androgen secretion, which is known to be ergogenic), each of which have been suggested to be abnormal in CF. Regardless of etiology, in CF, delayed biological maturation may influence the evolution of exercise capacity as patients age, especially with respect to sex hormone secretion, which is an important determinant of exercise capacity (Kindermann et al., 1982; Ogawa et al., 1992; Sheel et al., 2004; Molgat-Seon et al., 2018), and should thus be considered in exercise studies of people with CF. In particular, sex hormone secretion may influence the onset of puberty, rate of maturity, and in non-CF children, sex differences in exercise capacity exist even in pre-pubescent children. Whether the CF population exhibits more or less pronounced sex differences in exercise capacity is, however, presently unknown. Limited evidence suggests that adult and adolescent females with CF have lower physical fitness compared with males when matched for disease severity, however, it is presently unknown whether this is related to pathophysiology, behavior, or both, and whether these findings may extend to youth (Eisenstadt et al., 2016). Differences in the onset and rate of maturity are also likely to have social implications with regards to peer perceptions that influence engagement in physical activity and exercise.

### Treatment Strategies for Children and Adults With CF

The advent of CFTR modulator therapies has revolutionized the treatment of CF. These therapies can be broadly divided into CFTR potentiators, which improve ion and fluid conductance through the CFTR channel, and CFTR correctors, which aid in chaperoning mutated CFTR proteins during protein folding, thereby preventing endoplasmic reticulum (ER)-mediated degradation (Burgener and Moss, 2018; McElvaney et al., 2018; Habib et al., 2019). Currently approved therapies cover roughly 40% of individuals with CF, depending on age and mutation class (Cystic Fibrosis Foundation, 2017), and ongoing Phase 3 clinical trials of triple-combination therapies have the potential to extend that coverage to roughly 90% of people with CF (Davies et al., 2018; Holguin, 2018; Keating et al., 2018; Vertex Pharmaceuticals Incorporated, 2018a, b). Moreover, several ongoing trials are evaluating the use of CFTR modulator therapies in infants and toddlers (ranging from 0 to 24 months) with CF (Cystic Fibrosis Foundation, 2017). If successful, these trials will enable early initiation of highly effective CFTR modulator therapy, which has the potential to preserve pulmonary function at near-normal levels, and to arrest the annual decline in lung function that is currently characteristic of CF. This may, in turn, create a new “generation” of individuals with CF whose disease course is markedly different from the current CF population who may only have initiated CFTR modulator therapy after significant lung damage had already occurred. It is therefore postulated that such early intervention and preservation of lung function would substantially improve individuals’ exercise capacity by abrogating ventilatory dysfunction.

The age at which people with CF begin CFTR modulator therapy can have a profound effect on the disease course, and consequently, both morbidity and mortality. Most prominently, improvements in pulmonary function, as well as arresting the rate of decline in pulmonary function, greatly improve clinical outcomes, QoL, and the ability to exercise. The effects of CFTR modulator therapies on extra-pulmonary CF disease manifestations are less clear, and, in particular, it is still unclear whether CFTR modulators can improve diabetic status (Bellin et al., 2013; Tsabari et al., 2016; Thomassen et al., 2018; Kelly et al., 2019; Li et al., 2019). Nonetheless, CFTR modulator therapies have been shown to have a positive effect on weight gain and body mass index (Gelfond et al., 2017; Houwen et al., 2017; Gifford et al., 2018; Stallings et al., 2018), which might be beneficial for exercise capacity in malnourished or undernourished individuals with CF. Other therapies that help manage symptoms and sequelae of CF, such as inhaled hypertonic saline, airway clearance therapies, inhaled mucolytics, anti-inflammatories, and anti-microbials, may also vary in their use across the CF lifespan. For example, a recent investigation found that inhaled hypertonic saline could safely be used in infants with CF (Stahl et al., 2019), therefore enabling early intervention, which may aid in preserving lung function and slowing disease progression. It is clear, however, that when appropriate, the younger individuals with CF are when the treatment is initiated and the better these treatments are maintained, the better the clinical outcomes, morbidity and QoL (Davies et al., 2016; Rosenfeld et al., 2018). It also remains to be elucidated how the use of these therapies, and the age at which they are initiated, affects exercise capacity, physical activity levels and how to account for differences between individuals with CF in these regards when designing and analyzing exercise and physical activity studies. However, based on the clinical efficacy of these therapies, it is likely that early initiation of CFTR modulator therapy, effective symptom management and infection control, and regular airway clearance therapy all enhance the ability of individuals with CF to exercise. Thus, future studies should aim to determine how and to what extent these therapies are beneficial for exercise, and also control for the use of these therapies when classifying the CF population.

## Limitations in Comparing Children and Adults with CF

Overall, the progression of disease throughout the lifespan produces marked differences between children and adults with CF. It is therefore important to consider age-specific differences when interpreting exercise studies in these populations. Importantly, as those with CF age, they experience progressive declines in pulmonary function and increases in the incidence and prevalence of co-morbidities, both of which ultimately contribute to reduced exercise capacity. That is not to say that children with CF do not also possess reduced exercise capacity, however, the etiology of exercise impairment appears to be more complex in adults. Thus, when designing and conducting exercise studies in CF, it is important to ensure that appropriate descriptive measures which encompass these factors are employed, and that analyses control for, or account for, these factors. These types of analyses may require multivariate or mixed-model designs, thus it becomes important for researchers to include methodologists and biostatisticians in order to ensure both the design and analysis of exercise studies are appropriate and account for potential modifiers of exercise capacity that are unique to CF.

In addition to careful consideration of study design and analysis, the changes in pathophysiology across the lifespan in CF also highlights the need to conduct studies in both pediatric and adult populations including eras before and after initiation of CFTR modulator therapies, and not to simply use one population to draw conclusions about the overall CF population. It is clear that these populations are physiologically unique beyond simply being different in age. Therefore, understanding the differences in exercise physiology and pathophysiology in children vs. adults in CF may provide insight into how disease progression affects exercise intolerance. This information may aid in designing appropriate interventions and exercise prescription to reduce the decline in exercise capacity and improve patient health.

Presently, exercise training and testing are recommended for individuals with CF (Dwyer et al., 2011; Hebestreit et al., 2015; Radtke et al., 2017; Hebestreit et al., 2018; Urquhart and Saynor, 2018; Cox and Holland, 2019). However, specific recommendations for exercise prescription need to be developed further, and a recent systematic review on physical exercise training for CF concluded that the moderate quality of current evidence and small size, duration, and incomplete reporting of studies limits conclusions about the efficacy of physical exercise training for CF (Radtke et al., 2017). Future large, high-quality studies, including randomized controlled studies, are necessary to determine the optimal training components (including exercise modality, frequency, intensity, and duration) for individuals with CF (Radtke et al., 2017). In addition, we recommend future studies account for the factors discussed in this review, in order to better control for differences in age, disease progression, nutritional status, and other factors which may impact exercise capacity.

## Conclusion

In summary, disease progression in CF from childhood through adolescence to adulthood leads to progressively more complex exercise intolerance and provides a unique model to study differences in exercise capacity across the lifespan of individuals with CF. When undertaking exercise studies in CF, it is critically important to consider factors such as declining pulmonary function, increased chronic colonization by increasingly pathogenic and drug-resistant bacteria, increased frequency and severity of pulmonary exacerbations, endocrine comorbidities, nutritionally related factors, and modulator therapy. In particular, accounting for how these factors ultimately influence the ability to exercise is important to better understand exercise impairments in individuals with CF. As the expected lifespan with CF continues to increase with advancements in therapies, it is also important to better understand how these factors evolve over the lifespan as individuals with CF age, and to clarify how adult and pediatric populations differ. It is therefore important to conduct studies in both pediatric and adult populations to account for age-related differences and better understand *how* the evolution of CF disease impacts exercise (in)tolerance across the lifespan. Moreover, longitudinal studies of CF patients who begin CFTR modulator therapy early in life may also aid in understanding how arresting disease progression early in life affects exercise capacity over the lifespan of these patients.

## Author Contributions

All authors contributed to the manuscript conceptions, preparation, critical revision, and final editing, and approved the final manuscript before submission.

## Conflict of Interest

The authors declare that the research was conducted in the absence of any commercial or financial relationships that could be construed as a potential conflict of interest.
